# 

**Published:** 1912-01-20

**Authors:** 


### Appointments.

Mr. John, of Stoke-on-Trent, has been appointed radiographer to the Longton Cottage Hospital.

Mr. Guy de H. Dawson, M.R.C.S., L.R.C.P., has been appointed House Surgeon at the Evelina Hospital for Children.

Mr. Arthur H. Bostock has been appointed, subject to the approval of the Local Government Board, Medical Officer of Health to Chichester and Superintendent of the Isolation Hospital.

Dr. D. Bell, M.B., Ch.B. Edin., has been appointed House Surgeon at Swansea Hospital. Dr. T. Lewis Jones, who has served twenty years indoor service, was also appointed House Surgeon.

The vacancy for a Surgical Registrar at St. Bartholomew's Hospital has been filled by the appointment of Mr. Harold W. Wilson, M.B., F.R.C.S., hitherto demonstrator of anatomy in the Medical School. Mr. Wilson is also surgeon to out-patients at the Victoria Hospital for Children and assistant surgeon to the Cancer Hospital.

**Rules for Correspondents.**

Everyone who uses or wishes to help our Bureau of Information must be kind enough to observe the following rules:—

(1) Postal replies cannot be given. Exception will only be made in very special cases at the Editor's discretion.

(2) Queries or answers relating to different cases must be written, or preferably typed, on separate sheets of paper, and the writing must never be crossed.

(3) Questions and replies received not later than Wednesday will, as far as possible, be dealt with in our issue of the following Saturday week.

(4) Letters must have attached the name and address of the sender, with a pseudonym for publication if preferred.

(5) All applicants for insertion in our list of Approved Homes must send a registration fee of five shillings. The omission of this leads to delay in the insertion of names.

(6) *All communications* for this department *must be accompanied by the coupon* to be found at the bottom of the third page of the cover on the inside, and should be addressed to the Editor of the Bureau of Information, c/o The Hospital, 29 Southampton Street, Strand, London, W.C.

### Gifts and Bequests.

Mr. William Davies has left £200 to the Altrincham General Hospital.

Mr. Isaac C. Johnson, of Gravesend, has left £100 to the Gravesend Hospital.

Mr. John Malcolm Ludlow has left ten guineas to the Hospital for Women, Soho.

Mrs. Elizabeth Farquhar, of Portland Place, W., has bequeathed £1,000 to the Samaritan Free Hospital for Women, and £200 to the Royal Eye Hospital, Southwark.

Mr. Wm. Bell Readhead, of South Shields, has left £1,000 to the Ingham Infirmary.

Lady Alexandrina Cunliffe has left £100 to the Hospital for Diseases of the Chest, City Road.

The trustees of the late Mrs. C. M. Barnato have allocated £250 to Queen Charlotte's Hospital.

The After-Care Association has received £20 from the Clothworkers' Company and ten guineas from the Skinners' Company.

### Florence Nightingale Memorial.

We have received the following contributions since last week. They are for a statue, unless otherwise indicated : Sir James Affeck, M.D., £5 5s.; Nurse H. M. Walker, £1 1s.; Miss Wilson, £1; F. Smith, Esq., 10s. 6d. Per R. Orde, Esq., House Governor and Secretary Royal Victoria Infirmary, Newcastle-upon-Tyne : D. Drummond, Esq., M.D., £2 2s.; T. M. Allison, Esq., M.D., £1 1s.; H. B. Angus, Esq., F.R.C.S., £1 1s.; W. D. Arnison, Esq., M.D., £1 1s.; T. Beattie, Esq., M.D., £1 1s.; R. A. Bolam, Esq., M.D., £1s. 1s.; Horsley Drummond, Esq., M.D., £1 1s.; T. Gowans, Esq., M.B., Ch.B., £1 1s.; W. E. Hume, Esq., M.R.C.P., £1 1s.; J. W. Leech, Esq., M.D., £1 1s.; R. P. Ranken Lyle, Esq., M.D., £1; A. M. Martin, Esq., M.B., B.S., £1 1s.; Rutherford Morison, Esq., F.R.C.S., £1 1s.; Sir Thomas Oliver, M.D., £1 1s.; R. Orde, Esq., House Governor, £1 1s.; A. Parkin, Esq., M.D., £1 1s.; W. G. Richardson, Esq., F.R.C.S., £1 1s.; G. G. Turner, Esq., F.R.C.S., £1 1s.; J. D. Wardale, Esq., M.B., B.S., £1 1s.; S. S. Whillis, Esq., M.D., £1 1s.; Wm. F. Wilson, Esq., M.B., B.S., £1 1s.; Hamilton Drummond, Esq., M.B., B.S., 10s. 6d.; Miss Gurney, 10s. 6d.; J. W. Heslop, Esq., M.B., B.S., 10s. 6d.; R. J. Willan, Esq., F.R.C.S., 10s. 6d.; H. H. Markham, Esq., M.B., B.S., 5s.; W. J. Phillips, Esq., M.B., B.S., 5s.; Nursing Staff, £9 7s.; Total, £35. Per J. J. Barron, Esq., Sec.-Supt. Bradford Royal Infirmary : Fredk. Jas. Burnley, Esq., £2; Harry Behrens, Esq., M.A., J.P., £1 1s.; A. T. Parkinson, Esq., £1 1s.; Mrs. Whitehead, £1 1s.; J. J. Barron, Esq., 10s. 6d.; Reginald C. Lupton, Esq., 10s. 6d.; G. E. Priestman, Esq., 10s. 6d.; G. B. Stephenson, Esq., 10s. 6d.; Total, £7 5s.

Received by Mr. G. Q. Roberts : Sir W. G. Armstrong, Whitworth and Co., Ltd., £10 10s.; Nursing Staff London Hospital (in addition to £83 8s. 3d. already received), £8 15s.; Nursing Staff Royal Infirmary, Edinburgh, £7 11s.; Lord Decies, £5; Emily Lady Brown, £5; Prof. J. Rankine, K.C., £5; Nursing Staff N. Staffs Infirmary, Stoke-on-Trent, £5; "Sister," £5; Miss H. F. Cohen, £2 2s.; Wootton St. Lawrence Mothers' Union Members, £1 1s. 6d.; Miss Mabel Whiffen, £1 1s.; Dr. and Mrs. Jackson, £1 1s.; Miss Goodlatte (annuities), £1. Collection at Penmore Hospital, Chesterfield, 15s. 6d.; Miss Cicely Jackson, 10s. 6d.; Committee of Billericay and District Nursing Association, 10s. 6d.; Miss C. Jex Blake, 10s.; Miss J. Spencer, 5s.; F. K., 2s. 6d.; Miss W. Everingham, 2s. 6d.; Miss L. T. White, 2s.

